# IMPaCT: A Pilot Randomized Trial of an Intervention to Reduce Preterm Birth Among Non-Hispanic Black Patients at High Risk

**DOI:** 10.1089/heq.2022.0089

**Published:** 2022-12-19

**Authors:** Sarahn M. Wheeler, Kelley E.C. Massengale, Thelma A. Fitzgerald, Tracy Truong, Truls Østbye, Amy Corneli, Geeta K. Swamy

**Affiliations:** ^1^ Division of Maternal and Fetal Medicine, Department of Obstetrics and Gynecology, Duke University School of Medicine, Durham, North Carolina, USA.; ^2^ Diaper Bank of North Carolina, Durham, North Carolina, USA.; ^3^ Department of Biostatistics and Bioinformatics, Duke University School of Medicine, Durham, North Carolina, USA.; ^4^ Department of Family Medicine and Community Health, Duke University School of Medicine, Durham, North Carolina, USA.; ^5^ Department of Population Health Sciences, Duke University School of Medicine, Durham, North Carolina, USA.

**Keywords:** health inequity, intervention, preterm birth, racial disparity, randomized control trial

## Abstract

**Introduction:**

Preterm birth is a major cause of neonatal morbidity and mortality rate. Non-Hispanic black patients disproportionately experience preterm birth and nonadherence to evidence-based preventive measures. Interventions tailored to non-Hispanic black birthing individuals (NHBBIs) that address barriers to preterm birth preventions are urgently needed.

**Methods:**

Together with a community-engaged multidisciplinary stakeholder group, we developed an intervention to improve adherence to preterm birth preventions among black pregnant patients with prior preterm birth. The intervention included the following: (1) preterm birth prevention education, (2) an employment navigation toolkit, and (3) encouragement text messages. We piloted the intervention by recruiting self-identified non-Hispanic black patients at or before 20 weeks of gestation with a prior preterm birth and randomizing them to the intervention or an active control. The primary outcomes were feasibility and acceptability. Our secondary outcomes were preliminary efficacy based on birth outcomes, patient experience, and pregnancy-specific anxiety (PSA). Descriptive statistics, analysis of verbatim survey responses, Wilcoxon signed rank, and Fisher's exact were used to describe and compare quantitative and qualitative data.

**Results:**

We identified 53 individuals who met the inclusion criteria, 35 were reachable remotely and 30 were enrolled and randomized. More than 80% (*n*=26) were retained throughout the study, and 100% of participants identified at least one intervention component as helpful. In this small pilot, there were no detectable differences in adherence to preterm birth preventive recommendations. No difference in preterm births, other pregnancy, or patient experience outcomes was detected between the intervention and active control participants.

**Discussion:**

The intervention is feasible and acceptable. Larger, appropriately powered studies are needed to assess whether the intervention will decrease PSA and reduce preterm birth. This trial was registered with ClinicalTrials.gov (NCT04933812).

## Introduction

The rate of preterm birth (delivery before 37 weeks of gestation) is more than 50% higher among non-Hispanic black birthing individuals (NHBBIs) compared with all other racial and ethnic groups.^1^ The racial disparity in preterm birth is multifactorial, stemming from short- and long-term effects of individual, interpersonal, and systemic racism.^2^ This disproportionate impact of preterm birth in the black community is a leading cause of neonatal death and NHBBIs experience the highest rate of infant mortality rate.^1^ Neonates that survive preterm birth often have lifelong health problems and developmental delays.

Although preterm birth is an important health care issue, there are limited tools for predicting patients at high risk for preterm birth. The most important clinical predictor is a history of preterm birth, which is associated with a twofold increased risk of preterm birth in a subsequent pregnancy.^3^ The risk of recurrent preterm birth is fourfold higher for NHBBIs than their white counterparts.^4^ Due to their increased risk for preterm birth, NHBBIs with a prior preterm birth are an important target population for preterm birth prevention.

For patients with a prior preterm birth, guidelines encourage clinicians to evaluate a patient's obstetrical and medical history and then offer evidence-based recommendations tailored to optimize health and reduce the risk of recurrent preterm birth.^3^ Depending on the individual patient's history, evidence-based recommendations may include up to four major components, including: (1) lifestyle modifications (e.g., weight gain guidelines, nutrition, tobacco cessation, and exercise),^5^ (2) serial cervix lengths^6^ and/or cerclage,^7,8^ (3) supplemental progesterone,^9^ and (4) low-dose aspirin.^10,11^ Prior studies have shown that NHBBIs are at risk for nonadherence to some of these evidence-based preterm birth preventive measures.^12,13^

We developed the IMProving the Clinical encounter To enhance delivery of an Individualized Prematurity Prevention Plan (IMPaCT) intervention to mitigate patient-identified barriers and promote adherence to evidence-based preterm birth preventative recommendations. We pilot tested the IMPaCT intervention to determine its feasibility, acceptability, and preliminary efficacy.

## Methods

### Study design

We conducted a pilot randomized controlled trial to determine the (1) feasibility and acceptability of implementing IMPaCT (primary objectives) and (2) preliminary efficacy of IMPaCT by comparing adherence to the evidence-based recommendations for preterm birth prevention, delivery outcomes, and patient experience between patients assigned to the IMPaCT intervention versus the active control.

### Routine clinical practice

Consistent with guidelines from the American Congress of Obstetricians and Gynecologists (ACOG) and Society for Maternal Fetal Medicine (SMFM), all patients seen within our system who have a prior preterm birth receive counseling about reducing their risk of recurrent preterm birth. All patients are counseled lifestyle modifications, however, the other three major recommendations (i.e., serial cervix lengths and/or cerclage, supplemental progesterone, and low-dose aspirin) vary based on a patient's history. In our clinical practice, patients with a history of spontaneous preterm birth (i.e., idiopathic preterm labor or preterm rupture of membranes) are offered serial cervix length screening. While the monitoring plan may be individualized, most often cervix length screening is transvaginal, starts at 16–18 weeks, and includes monitoring every 2-week screening until 24 weeks. Patients whose history was strongly suggestive of cervical insufficiency or those who developed short cervix (less than 2.5 cm) are advised to consider cerclage.

All patients with a prior spontaneous preterm birth are also offered supplemental progesterone (vaginal or intramuscular).^5^ We recommend low-dose aspirin (81 mg/day) to all patients with a history of preterm birth in the setting of preeclampsia or other risk factors as outlined in the U.S. Preventive Service Task Force Guidelines.^14^

### Participants

**FIG. 1.**
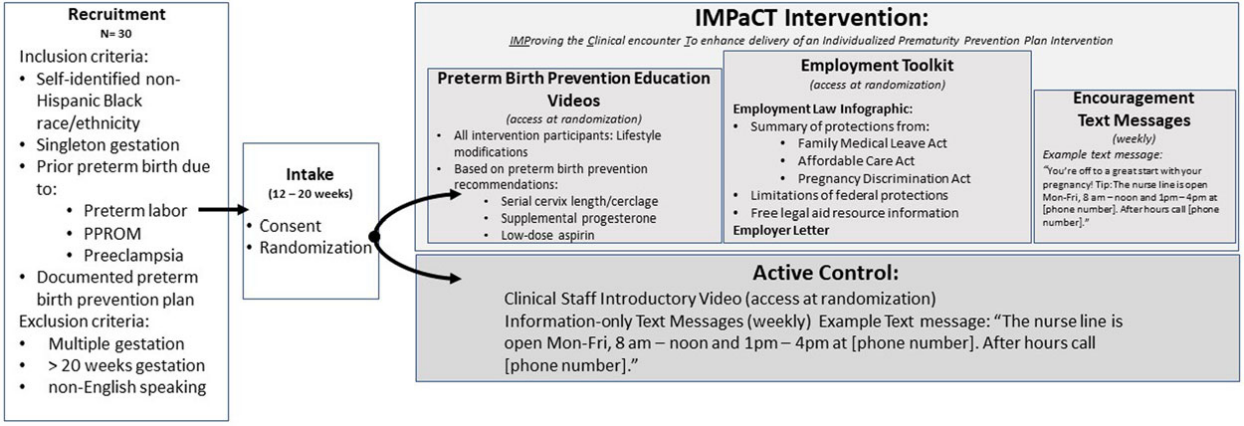
Participants' flow through the IMPaCT intervention randomized controlled trial. IMPaCT, IMProving the Clinical encounter To enhance delivery of an Individualized Prematurity Prevention Plan.

Participants were recruited after their initial prenatal visit, counseling about preterm birth prevention with a high-risk obstetric clinician, and documentation of their care plan. The IMPaCT intervention was developed as an adjunct to routine high-risk obstetrical care. Participation in the randomized trail did not affect routine clinical care or counseling. The clinical staff was blinded to the participants' randomization and all participants were counseled as per their clinician's usual clinical practice. Participants' flow through the study protocol including recruitment, randomization, and the IMPaCT intervention or active control study arms is summarized in Figure 1. The study was approved by Duke University's Institutional Review Board (Pro00103922).

We recruited participants from a single high-risk obstetrical clinic at a tertiary care center in the American southeast. Patients were eligible if they self-identified as non-Hispanic black race and ethnicity and had a current singleton gestation with a prior spontaneous (i.e., due to idiopathic preterm labor, preterm prelabor rupture of membranes) medically indicated preterm birth (i.e., due to preeclampsia). We included patients with a history of spontaneous and medically indicated preterm birth because both groups are at increased risk for future preterm birth, there are guidelines for preterm birth prevention targeted at both groups, and some patients have histories that include risk factors for spontaneous and medically indicated preterm birth. Patients beyond 20 weeks of gestation were excluded because many preterm birth preventive measures should be initiated by that gestational age.

Patients with multiple gestations were also excluded because many of the evidence-based preterm birth preventive interventions are not proven effective in this setting. Non-English proficient patients were also excluded because the intervention materials are currently only available in English.

We identified potential participants meeting the eligibility criteria by reviewing the electronic health record (EHR). Due to the COVID 19 pandemic, we conducted all recruitment and intervention delivery activities remotely through telephone. After consent and enrollment, we conducted study activities throughout participants' pregnancies, until their postpartum visits at 4–12 weeks following delivery.

### Randomization

After enrollment and consent, participants were randomized using a randomization table embedded generated by the statistical team and embedded into the REDCap™ data software. Randomization was one to one, and stratified on the three major recommendations that vary based on patient history (i.e., serial cervix lengths and/or cerclage, supplemental progesterone, and low-dose aspirin).

### Study arms

#### IMPaCT intervention

The IMPaCT intervention was developed in partnership with a diverse group of stakeholders including non-Hispanic black patients who had experienced preterm birth, obstetrical clinicians, and administrative leaders in the high-risk obstetrical clinic and community-led organizations. The stakeholders reviewed data from our previously conducted qualitative studies.^15,16^ During these prior qualitative studies, NHBBIs with a history of preterm birth identified inconsistent clinical instructions and employment obligations as important barriers, and encouragement as a potential facilitator, to preterm birth prevention adherence. IMPaCT was designed to address each of these themes.

We developed preterm birth prevention education to address the need for consistent clinical instructions. Patients often receive care from multiple clinical providers who are part of a group practice during their prenatal appointments. Different clinicians may describe preterm birth preventions using slightly different terminology. During the qualitative studies, patients reported that hearing different explanations for preterm birth preventions lead to fear and distrust.^15^ We developed a series of narrated educational PowerPoint™ video presentations to explain terminology in greater detail than is often possible during the brief time allocated in a clinical visit.

Each presentation focused on one of the following four major evidence-based recommendations to prevent preterm birth recurrence: (1) lifestyle modifications, (2) serial cervix lengths and cerclage, (3) progesterone therapy, and (4) low-dose aspirin. Immediately after randomization, IMPaCT intervention participants were emailed a link to the PowerPoint video presentation(s) based on the evidence-based recommendation that they received during their routine clinical visit. Thus, each IMPaCT participant received at least one (i.e., lifestyle modifications) and up to four educational presentations.

To address employment barriers, we partnered with the Duke Law School Health Justice Clinic and developed a toolkit that includes a patient-facing infographic about employment laws in pregnancy and a letter for employers. The infographic includes plain-language summary of the federal and North Carolina state laws that govern employment during pregnancy. The plain-language summaries include details about specific protections such as 12 weeks of job protection during medical leave, when these laws do (and do not) apply, and phone numbers to contact free legal aid services.

The employment toolkit includes a form letter given to patients that they can in turn give to their employer. The letter informs the employer that their employee (i.e., the participant) is receiving care for a high-risk pregnancy and this care may require close monitoring with frequent appointments. Participants were counseled that this letter is intended to empower them with language to start a conversation with their employer about their high-risk pregnancy and the potential need for more flexible hours to attend clinic visits.

We addressed requests for encouragement identified in the qualitative studies by sending SMS text messages to IMPaCT intervention participants that included a basic pregnancy fact and a note of encouragement. The text messages started at the initiation of the intervention and continued weekly until delivery. Participants who reached 28 weeks of gestation also received a formal letter of encouragement mailed to their home. Text messaging was used due to ease of message distribution and the wide availability among the target patient populations. The text messages were distributed from a generic research messaging account. Although participants consistently reported a desire for encouragement, our data were unclear about from whom participants wanted to receive encouragement. Therefore, we also included the provider encouragement letter to provide more personalized encouragement directly from a clinician known to the participants (study primary investigator).

#### Active control

Participants in the active control received guideline-consistent, evidence-based preterm birth preventative care recommendations and education from their clinical providers as part of their routine clinical care. Participants randomized to the active control received (1) a PowerPoint presentation that provided general information about clinic staff without education about the recommendations for preterm birth prevention, and (2) weekly texts that contained only basic pregnancy facts without encouragement messages. Active control participants were not given the employment toolkit.

### Primary outcome measures

The primary outcomes of the pilot study were feasibility and acceptability of the IMPaCT intervention. *Feasibility* was evaluated using recruitment and retention metrics. Based on recruitment for our prior qualitative work,^15,16^ we anticipated that 30% of eligible participants approached would enroll in the study, and 80% would participate until the end of study follow-up. *Acceptability* was evaluated in participants randomized to the intervention arm through an open-ended telephone survey at the conclusion of the study. During the telephone survey, we asked participants to identify the most and least helpful intervention components, suggest strategies to improve the intervention, and rate the amount of time spent on the intervention (too much, too little, or about right). Participant responses were recorded verbatim by the interviewer.

### Secondary outcome measures

Our secondary outcomes were adherence to preterm birth prevention recommendations advised by their clinical provider, delivery outcome (e.g., term vs. preterm delivery), and the participant's experience with clinical care, social support, and pregnancy-specific anxiety (PSA).

At initial enrollment, we collected demographics, and obstetric and medical history based on EHR review. By reviewing the participant's EHR and self-report, we measured adherence to lifestyle modifications using the Institute of Medicine weight gain guidelines; adherence to cerclage or cervix length screening based on documented receipt of cerclage or adherence to cervix length ultrasounds every 2 weeks from 16 to 24 weeks of gestation; progesterone adherence based on EHR documentation of supplemental progesterone receipt, and low-dose aspirin adherence based on self-report in the EHR. We abstracted pregnancy outcomes including term versus preterm delivery and gestational age at delivery from the EHR. We also abstracted diagnosis of preeclampsia or gestational hypertension, the number of antenatal triage evaluations, antepartum inpatient admission, and the length of hospital admission following delivery.

We quantified patient experience through surveys that measured the participants' perception of their care experience, social support, and PSA. We postulated that the education provided through the IMPaCT intervention might improve participants' perception of care and reduce their PSA over the course of the intervention. We measured participant's care experience with the Interpersonal Processes of Care (IPC) survey. The IPC survey is an 18-question validated instrument designed to evaluate a patient's perceptions of interactions with medical providers across patients of diverse racial and ethnic backgrounds.^17^ Each of the 18 questions map to one of the following three domains: communication, decision-making, and interpersonal style. Within the communication domain, participants are asked to rate their provider's clarity, ability to solicit concerns, and whether the clinician explained test results.

The decision-making domain mapped to questions about whether the provider involved the patient in developing the plan of care or asked about barriers to the treatment plan. The interpersonal style domain mapped to questions about communication and discrimination. Each of the 18 questions is answered on a Likert scale ranging from never to nearly always.

We measured social support using the Maternal Social Support Scale (MSSS), a six-question questionnaire designed to be a screening tool for perceived social support in pregnancy.^18^ The MSSS asks participants to respond to a series of statements about support from friends, family, and their husband/partner. Each question is answered on a five-point Likert scale from never to always.

We measured PSA using the PSA scale.^19^ PSA is a patient-centered outcome measured by a four-question survey. The survey asks participants how often they have felt *anxious*, *concerned*, *afraid*, or *panicky* about their pregnancy over the past week. Participants rate the frequency of each emotion on a Likert scale ranging from “not at all” (score=1) to “very much” (score=5). The numerical score for each emotion is averaged to generate a final score.

The IPC-18 and MSS were distributed at intake (12–20 weeks of gestation), third trimester (24–30 weeks), and at the protocol conclusion (∼1 month after delivery). The PSA was administered at intake and third trimester because the questions are specific to the pregnant state.

### Statistical analyses

We used descriptive statistics to describe the participant demographics and pregnancy outcomes in all participants. Feasibility was determined among participants randomized to the intervention using descriptive statistics. Acceptability was measured among participants randomized to the intervention based on evaluation of verbatim responses to a telephone-administered open-ended survey by the study team. Illustrative quotes were selected by the study team to highlight the survey findings. We measured preliminary efficacy by comparing adherence to preterm birth preventative recommendations, changes in IPC-18, MSS, and PSA between participants in the intervention and active control groups using the Wilcoxon signed rank tests for continuous variables and Fisher's exact testing for categorical variables. Analyses were conducted with SAS 9.4 (SAS Institute, Inc., Cary, NC, USA).

## Results

### Enrollment and participant characteristics

Between February and August 2020, we identified 53 individuals who met the inclusion criteria. Of these individuals, 18 (34%) were unreachable remotely and therefore not approached for enrollment, 5 (9%) were uninterested, and 30 were enrolled and randomized. We randomized 14 participants to the IMPaCT intervention and 16 to the active control arm. There were no clinically or statistically significant differences in age, number of prior preterm births, or body mass index. Other baseline health conditions associated with preterm birth, including chronic hypertension, diabetes, and tobacco use, were uncommon and also similar across the groups. Demographic characteristics are summarized in Table 1.

**Table 1.** Participant Characteristics

Characteristic	Active control (*N*=16)	IMPaCT (*N*=14)
Age (mean, SD)	32.1 (7.0)	30.9 (5.3)
No. of prior preterm births (median, IQR)	1.5 (1, 2)	1 (1, 2)
Insurance status		
Public	12 (75)	11 (79)
Private	3 (19)	3 (21)
None	1 (6.2)	0 (0)
Body mass index at prenatal care intake (mean, SD)	33.9 (8.9)	37.3 (9.0)
Chronic hypertension	2 (12.5)	0
Pregestational diabetes	0	1 (7.1)
Tobacco use at any time in pregnancy	1 (6.2)	1 (7.1)
Preterm birth preventative recommendations		
Lifestyle modifications, *n* (%)	16 (100)	14 (100)
Cervix length/cerclage, *n* (%)	11 (69)	9 (64)
Progesterone, *n* (%)	8 (50)	8 (57)
Low-dose aspirin, *n* (%)	13 (81)	11 (79)

All data are reported as *n* (%) unless detailed otherwise.

IMPaCT, IMProving the Clinical encounter To enhance delivery of an Individualized Prematurity Prevention Plan; IQR, interquartile range; SD, standard deviation.

### Primary outcomes

We exceeded our target and recruited 30 of the 53 (57%) eligible participants. Although 53 patients were potentially eligible, only 35 could be reached for remote recruitment, and thus, we recruited 86% (30/35) of participants who were reachable for remote enrollment. Throughout the study, only one participant (3%) who delivered at 22 weeks and suffered a neonatal death was completely lost to follow-up. In addition, three patients (10%) remained connected to the study staff throughout the study, yet did not complete all of the study assessments. The remaining 26 participants (87%) completed the study (Table 2).

**Table 2.** Feasibility and Acceptability: Metrics, Targets, and Pilot Results

Measure	Metric	Pilot study results (%)
Feasibility	Recruitment: % of eligible patients recruited	57
	Retention: % of participants retained in study	87
Acceptability	% who rated at least one intervention component as “helpful”	100
	% who rated any intervention component as “unhelpful”	0

Twelve out of the 14 (86%) participants randomized to the intervention completed the telephone survey. Overall, the pilot intervention participants were overwhelmingly positive about their experience, 86% responded that the IMPaCT intervention met their needs and more than 90% of the interviewed participants reported that the amount of time spent on the intervention felt “about right.” All 12 (100%) participants reported that they found at least one intervention component helpful, and that no intervention component was unhelpful.

When asked about the employment toolkit, participants said they found the infographic and employer letter informative and useful. One participant commented, “[I] didn't know I had those rights.” Another participant similarly stated, “[I] felt vulnerable. It was good to know I had rights.” Participants also described using the employment toolkit. A participant commented, “Shortly after I received it, I used this letter and found the information helpful.” Participants also responded positively to the educational videos. One participant commented, “I was more engaged in doctor-patient conversation, as I knew a lot more than before.”

Four participants reported that the SMS text messages were their favorite part of the intervention. One commented, “They were exciting. It was like a countdown each week that I received them. Also, they made me feel good when it said things like, ‘you are doing a great job’. [It] made me feel good about myself and how I was looking after my baby and myself.”

### Secondary outcomes

We observed a similar number of preterm births (<37 weeks) in both study arms, and the number of antepartum triage evaluations was equivalent between groups (Table 3). Although not statistically different in the small pilot, participants randomized to IMPaCT had fewer cases of preeclampsia, fewer antepartum admissions, and shorter hospital stays after delivery along with a lower mean gestational age and birthweight at delivery.

**Table 3.** Adherence and Pregnancy Outcomes

	Active control (*N*=16)	IMPaCT (*N*=14)	*p*
Preterm birth preventative recommendation adherence (adhered/recommended, percent)^a^			
Lifestyle modifications			
IOM weight gain guidelines	3/16 (18.8%)	1/14 (7.1%)	0.602
Tobacco cessation	0/1 (0%)	1/1 (100%)	1.000
Cerclage/serial cervix lengths			
Cerclage	1/1 (100%)	4/4 (100%)	—
Serial cervix lengths	9/10 (90%)	4/4 (100%)	1.000
Low-dose aspirin	6/14 (42.9%)	3/12(25%)	0.429
Pregnancy outcomes			
Preterm birth/gestational age outcomes			
Preterm delivery <37 weeks, *n* (%)	8 (50)	7 (50)	1.000
Gestational age at delivery in weeks, mean (SD)	35.7 (3.5)	34.0 (6.1)	0.338
Maternal outcomes			
Preeclampsia/gestational hypertension, *n* (%)	4 (25)	1 (7.1)	0.336
No. of antepartum triage evaluations, median (IQR)	3 (2, 4)	3 (2, 6)	0.532
Antepartum hospital admission, *n* (%)	5 (31.3)	3 (21.4)	0.689
Length of delivery admission in days, median (IQR)	3 (2, 3)	2 (2, 3)	0.286
Neonatal outcomes			
Birthweight in grams, mean (SD)	2607.9 (750.6)	2442.5 (1255.9)	0.660
Neonatal intensive care unit admission, *n* (%)	4 (25)	5 (35.7)	0.694

^a^ Adherence to progesterone was not captured due to abrupt switch from clinic to at-home administration in the setting of the COVID-19 pandemic.

IOM, Institute of Medicine.

In both groups, adherence to Institute of Medicine weight gain guidelines and low-dose aspirin was low (ranging from 0% to 43%), while adherence to cerclage and serial cervix lengths was high (ranging from 90% to 100%). Of the two participants who used tobacco, the participant randomized to the IMPaCT intervention reported quitting tobacco and the control group participant was still smoking at the conclusion of the study. We were unable to capture adherence to supplemental progesterone because dosing administration switched abruptly from majority clinic-based administration to home injections due to the COVID-19 pandemic.

We observed very similar responses to IPC-18, MSS, and PSA between groups at intake. Furthermore, there were no statistically significant differences between groups in these measures at the third trimester or postpartum assessments. Three participants in the IMPaCT intervention arm and one participant in the control arm delivered before the third trimester survey instruments. There were also no statistically significant differences in IPC-18, MSS, or PSA, comparing study arms. Yet, we noted that patients randomized to the intervention rated their overall care experience at the conclusion of the study period worse than participants in the active control on the IPC-18. There was a decrease in social support noted among participants in the active control, while there was a small increase among the IMPaCT group. In addition, PSA was higher in the IMPaCT group at baseline, while the means were similar at the third trimester (Table 4).

**Table 4.** Secondary Quantitative Measure (Mean, SD)

	Active control (*N*=15)	IMPaCT (*N*=11)	*p*
IPC-18 at protocol completion (Description: validated 18-question instrument that measures patient care experience during clinical visits in four domains. Scores for each domain range from 1 to 5 on a Likert scale)			
Communication domain			
Lack of clarity (higher scores are negative, denoting increased lack of clarity from clinicians)	1.6 (1.1)	1.6 (0.6)	0.486
Elicits concern (higher scores are positive, denoting the clinician-elicited concerns more often)	4.7 (0.6)	4.6 (0.6)	0.573
Explain results (higher scores are positive, denoting the clinician-explained results more often)	4.6 (0.7)	4.5 (0.6)	0.532
Decision-making domain			
Decision-making (higher scores are positive, denoting more collaborative decision-making between the clinician and patient)	4.8 (0.4)	4.4 (1.1)	0.293
Interpersonal style domain			
Emotional support (higher scores are positive, denoting more emotional support from the clinician)	4.8 (0.4)	4.7 (0.5)	0.500
Discrimination due to race/ethnicity (higher scores are negative, denoting increased discrimination from clinicians)	1.3 (0.8)	1.5 (0.7)	0.261
Disrespectful to office staff (higher scores are negative, denoting increased disrespectful behavior from clinicians toward office staff)	1.1 (0.5)	1.2 (0.3)	0.428
MSSS (Description: six-question questionnaire answered from 1 to 5 on a Likert scale, higher scores are positive and denote increased support)			
MSS at intake	28.1 (2.0)	27.1 (3.4)	0.433
MSSS at protocol completion	28.3 (2.0)	26.9 (3.1)	0.194
Change in MSSS	−1.4 (2.4)	0.1 (1.5)	0.071
PSA at 28 weeks (Description: frequency of four emotions rated from 1 to 5 on a Likert scale, higher scores are negative and denote increased anxiety)			
PSA at intake	2.41 (0.9)	2.57 (1.0)	0.722
PSA at third trimester	2.33 (0.6)	2.34 (0.9)	0.958
Change in PSA	−0.17 (0.6)	−0.23 (1.1)	1.000

IPC, Interpersonal Process of Care; MSSS, Maternal Social Support Scale; PSA, pregnancy-specific anxiety.

## Discussion

In our pilot randomized trial, we exceeded our feasibility and acceptability goals for the IMPaCT intervention. Participants were enthusiastic about the IMPaCT intervention components as evidenced by their willingness to enroll and engage with the intervention, even during the start of a global pandemic. Furthermore, the enthusiastic comments during the telephone survey suggest that IMPaCT is promising.

Adherence to preterm birth preventive therapies, birth outcomes, care experience, and social support were similar between groups. Although not statistically significant, the decrease in PSA was greater among participants randomized to the IMPaCT intervention. Our findings suggest that the IMPaCT intervention is a feasible and acceptable strategy worthy of an adequately powered trial to determine the efficacy to address the disproportionate preterm birth rate in the non-Hispanic black community.

It is unlikely that there is one solution or a single intervention that will eradicate long-standing, multifactorial disparities such as preterm birth. The disproportionate preterm birth rate among NHBBIs stems from racism and inequities at the societal, institutional, and individual levels.^20–22^ Appropriately, there is growing attention on prenatal care redesign to reduce preterm birth among NHBBIs. Group prenatal care^23–25^ and the use of doulas^26,27^ are examples of care redesigns that have demonstrated some success. However, widespread adoption is limited due to implementation challenges, particularly in states such as North Carolina, where doula services are not covered by Medicaid. Policy changes that allow bold care redesigns will take time in the current political environment. In contrast, the IMPaCT intervention components are low cost and readily scalable within both the health care and community contexts.

Although the study was underpowered to detect any differences in clinical outcomes, we did not observe any trends to suggest improved adherence to preterm birth preventions, fewer preterm deliveries, or improved pregnancy outcomes. Despite no signal to suggest clinical improvement, one promising observation was the decrease in PSA among intervention participants. Anxiety due to lived experiences with racism has long been considered a potential etiology of the very high preterm birth rate among NHBBIs.^28–37^ Yet, interventions designed to reduce psychosocial stress during pregnancy have yielded minimal success.^23,38–43^ One major limitation is the lack of understanding of which types of stress (i.e., acute or chronic) are most important in the pathogenesis of preterm birth.

Roesch et al^19^ conducted a prospective cohort study of more than 400 pregnant people from diverse racial groups assessing dispositional, perceptual, and environmental stress measures with birth outcomes. After controlling for medical comorbidities, the only stress measure that was associated with birth outcomes was PSA. PSA measures how often a pregnant person felt *anxious*, *concerned*, *afraid*, or *panicky* about her pregnancy in the last week. PSA tends to decrease between the second and third trimester; yet a more profound decrease is associated with later gestational age at delivery. In addition, NHBBIs are at increased risk for elevated PSA.^19,44,45^

During our pilot study, we observed a decrease in PSA compared with the active control that did not reach statistical significance, yet suggests the need for a larger, appropriately powered randomized trial. Published data suggest that a 0.17 decrease in PSA translates to an additional week of gestation^19^; therefore, we believe that the 0.23 reduction in PSA among the intervention participants suggests that IMPaCT may be a clinically meaningful effect.

Although many of our findings are promising, we also noted that patients randomized to the IMPaCT intervention reported a less favorable care experience based on the IPC-18. The difference did not reach statistical significance; however, the trend was consistent across all domains of the IPC-18. Based on the positive feedback during the telephone surveys and reduction in PSA, we suspect the less favorable IPC-18 scores may be because that the IMPaCT intervention raised patient expectations for their care.

There are also several limitations that must be considered when evaluating these data. First, our data are from a small pilot at a single institution, limiting the generalizability of our findings. In addition, our data on acceptability are based on a telephone survey conducted by an interviewer who was part of the study, and thus, responses may have been biased by socially desirable responses.

## Conclusion

The high participant engagement in the IMPaCT intervention, among a patient cohort more often affected by barriers to adherence, suggests that IMPaCT is a feasible, acceptable, and patient-centered intervention for non-Hispanic black patients at highest risk for preterm birth, which provides evidence for implementing a future efficacy trial.

## Abbreviations Used

EHR: electronic health record
IMPaCT: IMProving the Clinical encounter To enhance delivery of an Individualized Prematurity Prevention Plan
IOM: Institute of Medicine
IPC: Interpersonal Processes of Care
IQR: interquartile range
MSSS: Maternal Social Support Scale
NHBBI: non-Hispanic black birthing individuals
PSA: pregnancy-specific anxiety
SD: standard deviation
